# Practical Notes and Formulæ

**Published:** 1887-01-01

**Authors:** 


### PRACTICAL NOTES AND FORMULÆ.

**Superior Carminative**—

| | | |
|---|---|---|
| R. | Tinc. cardaman | 4 ounces. |
| | Syrup ginger | 1½ ounces. |
| | Tinc. capsicum | 1 drachm. |
| | Tinc. nux. vomica | 1 drachm. |
| | Ess. peppermint | 1 drachm. |
| | Bi-carb. soda | 1 drachm. |

M. Dose one teaspoonful as a stomachic—useful in flatulent dyspepsia and other forms of indigestion.—R. C. WORD.

**A Good Styptic**—

| | | |
|---|---|---|
| R. | Collodion | 100 parts. |
| | Acid carbolic | 10 parts. |
| | Tannin | 5 parts. |
| | Benzoic acid | 5 parts. |

Shake the bottle well to insure the complete solution of all the drugs. This is an excellent protection to abraded parts and to coming boils.—*Ec. Med. Journal.*

| | | |
|---|---|---|
| R. | Boric acid | ʒ iij. |
| | Biborate of soda | ʒ viii. |
| | Glycerine | ℥ i. |

M. Ten to twenty drops, or even teaspoonful, at a dose constitute a valuable remedy in cystitis. Add the whole to a pint of water and you have the best antiseptic any reasonable man should have use for as external application in wounds, etc.—*Ibid.*

**Colic in Children.**—Condie (*Cleveland Medical Gazette*) recommends the following to prevent the return of colic in children:

| | | |
|---|---|---|
| R. | Ext. hyoscyam | grs. iv. |
| | Magnes. calcin | grs. xxiv. |
| | Pulv. ipecac | grs. ij. |

M. Ft. pulv. xii, et sig.—One every three hours.—*Medical Summary.*

**An Application for Pruritus Vulvæ**—

| | | |
|---|---|---|
| R. | Glycerite of starch | 30 parts. |
| | Zinc oxide | 6 parts. |
| | Potassium bromide | 10 parts. |
| | Ext. Indian hemp | 2 parts. |

Precede the application by a hot hip bath.—*N. Y. Med. Journal.*

**Hypodermic Solution of Quinine.**—Where it is necessary to administer quinine subcutaneously, the following formula is recommended by Dr. S. Burt as being as little irritating as possible:

℞. Quiniæ bisulphatis ʒ j.

Acidi borici gr. ij.

Morphinæ sulphatis gr. $\frac{1}{4}$.

Aquæ distillatæ ℥ j.

Sig.— For hypodermic use. One drachm contains seven and a half grains of quinine.— *Med. Reporter.*

**Cutaneous Anodyne.**— Dr. R. G. Gough, in the *Virginia Medical Monthly*, recommends the following prescription as one of the best he has ever found as a lotion for itching cutaneous surfaces, whether the skin is broken or not:

℞. Sodæ biborat ʒ j.

Acid carbol gtt. xv.

Glycerin ℥ j. M.

Sig.— Apply as lotion with camel's hair brush, or by dropping from bottle on the itching surfaces.

**Treatment of Acute and Chronic Nasal Catarrh.**— Dr. H. Marks, of St. Louis, writes us as follows (*Therapeutic Gazette*):

"I have a very good prescription for a snuff for acute or chronic catarrh, also traumatic rhinitis and acute coryza, especially when accompanied with pain of the nasal nerve. This formula effects a cure in twelve hours. It can be used as a snuff by the patient himself or in a powder blower.

℞. Cocaine hydrochl gr. x.

Ol. eucalyptus gr. iii.

Iodoform ʒ i.

Milk sugar, ad. q. s ℥ i. M.

Ft. triturate (snuff).

Sig.— Use every two or three hours. When relieved use two or three times a day.

Another formula which I have found of service, is a modification of that recommended by Beverly Robinson, which is as follows:

℞. Pul. fol. belladonnæ gr. xx.

Cocaine muriate gr. iv.

Ol. rosæ gtt. i.

Pulv. gum acaciæ, ad q. s ℥ ss. M.

Ft. triturate (snuff).

Sig.— Use with the powder-blower for anterior and posterior nares.

**Chionanthus Virginica in Jaundice.**— Henning employs the drug, associated with podophyllin or leptandrin, as follows:

℞. Extr. of chionanthus f℥ i.

Podophyllin fʒ i.

Acet. of potassium fʒ ss.

Water f℥ iv. M.

S.—One teaspoonful every three or four hours.

